# Application of molecular SERS nanosensors: where we stand and where we are headed towards?

**DOI:** 10.1007/s00216-020-02779-2

**Published:** 2020-07-16

**Authors:** Izabella J. Jahn, Anna Mühlig, Dana Cialla-May

**Affiliations:** 1 Leibniz Institute of Photonic Technology, Member of the Leibniz Research Alliance “Leibniz Health Technologies”, Albert-Einstein-Str. 9, 07745 Jena, Germany; 2 Center for Sepsis Care and Control Jena, Jena University Hospital, Kollegiengasse 10, 07743 Jena, Germany; 3 Institute of Physical Chemistry and Abbe Center of Photonics, Friedrich Schiller University, Helmholtzweg 4, Jena, Germany; 4 Center of Applied Research, InfectoGnostics Research Campus Jena, Philosophenweg 7, 07743 Jena, Germany

**Keywords:** Surface-enhanced Raman spectroscopy (SERS), Molecular nanosensors, Cellular microenvironment, Environmental sensing

## Abstract

Molecular specific and highly sensitive detection is the driving force of the surface-enhanced Raman spectroscopy (SERS) community. The technique opens the window to the undisturbed monitoring of cellular processes in situ or to the quantification of small molecular species that do not deliver Raman signals. The smart design of molecular SERS nanosensors makes it possible to indirectly but specifically detect, e.g. reactive oxygen species, carbon monoxide or potentially toxic metal ions. Detection schemes evolved over the years from simple metallic colloidal nanoparticles functionalized with sensing molecules that show uncontrolled aggregation to complex nanostructures with magnetic properties making the analysis of complex environmental samples possible. The present article gives the readership an overview of the present research advancements in the field of molecular SERS sensors, highlighting future trends.

## Introduction

Surface-enhanced Raman spectroscopy (SERS) is an attractive tool in analytical sciences, biology, biomedicine etc. It can quantitatively estimate low molecular weight substances in complex biofluid matrices, detect tumour margins or identify bacterial cells to name only a few examples from the huge variety of application scenarios mentioned in literature [1–6]. The method relies on the inelastic light scattering, known as Raman scattering, and it provides information on the vibrational modes of the investigated molecules and gains its sensitivity from the presence of enhanced electromagnetic field created in the proximity of metallic nanoparticles excited under resonance conditions. The electromagnetic and chemical enhancement mechanisms are the two underlying processes that lead to the enhanced Raman signal [7].

In SERS-based detection schemes, four main concepts are known: (1) label-free or direct SERS sensing is mostly applied and relies on the high affinity of the target analyte towards the metallic surface allowing for a SERS-based detection even in complex matrices [1, 4]. As analytes, mostly low molecular weight substances, e.g. drugs, metabolites, organic pollutants or small biomolecules, are targeted by label-free SERS approaches in complex matrices such as biofluids or surface water sources. (2) In a further sensing strategy, SERS tags which are composed of plasmonic nanoparticles (NPs), Raman reporter molecules and recognition elements are applied as stable and bright labels in e.g. DNA detection schemes or immunoassays [2, 4, 8]. (3) To allow in situ measurements of contaminated surfaces or buried interfaces, SERS-active nanoparticles are placed directly on the regions of interest creating the concept of shell-isolated nanoparticle-enhanced Raman spectroscopy (SHINERS) [9]. (4) Finally, molecular SERS sensors are described as SERS-active nanostructures modified with a sensing molecule which changes its molecular structure and/or orientation towards the metallic surface upon the interaction with a small molecule or ion [10]. Thus, the SERS signal of the sensor molecule is changed as function of the concentration of the target analyte. Moreover, target analytes are interacting in a specific manner with the sensor structure and could be enriched also from complex matrices.

Within this trend article, we summarize recent research results on the application of molecular SERS nanosensors in analytical science. Small molecules, such as reactive oxygen species (ROS), seldom show a significant or strong Raman signal. In the case of one-atomic ions, no vibrational Raman signal is observed as these species do not vibrate. Using metallic NPs functionalized with Raman-active molecular species capable of interacting with these targets in a highly specific manner makes the impossible possible, namely, i.e. the determination of intercellular pH value or the determination of Cu^2+^ concentration in complex samples. The detection is based on the fact that upon a chemical reaction, the molecular structure of the sensing molecule is changed resulting in the intensity change or position shift of the marker band or appearance of new Raman modes. Moreover, due to the formation of complex structures, the orientation of Raman marker modes towards the metallic surface is changed and in accordance with the surface selection rules, the SERS fingerprint region undergoes modifications. First, we will discuss the sensing of the cellular microenvironment and within the second part, we will report on molecular SERS nanosensors in environmental monitoring.

## Cellular microenvironment sensing

**Fig. 1:**
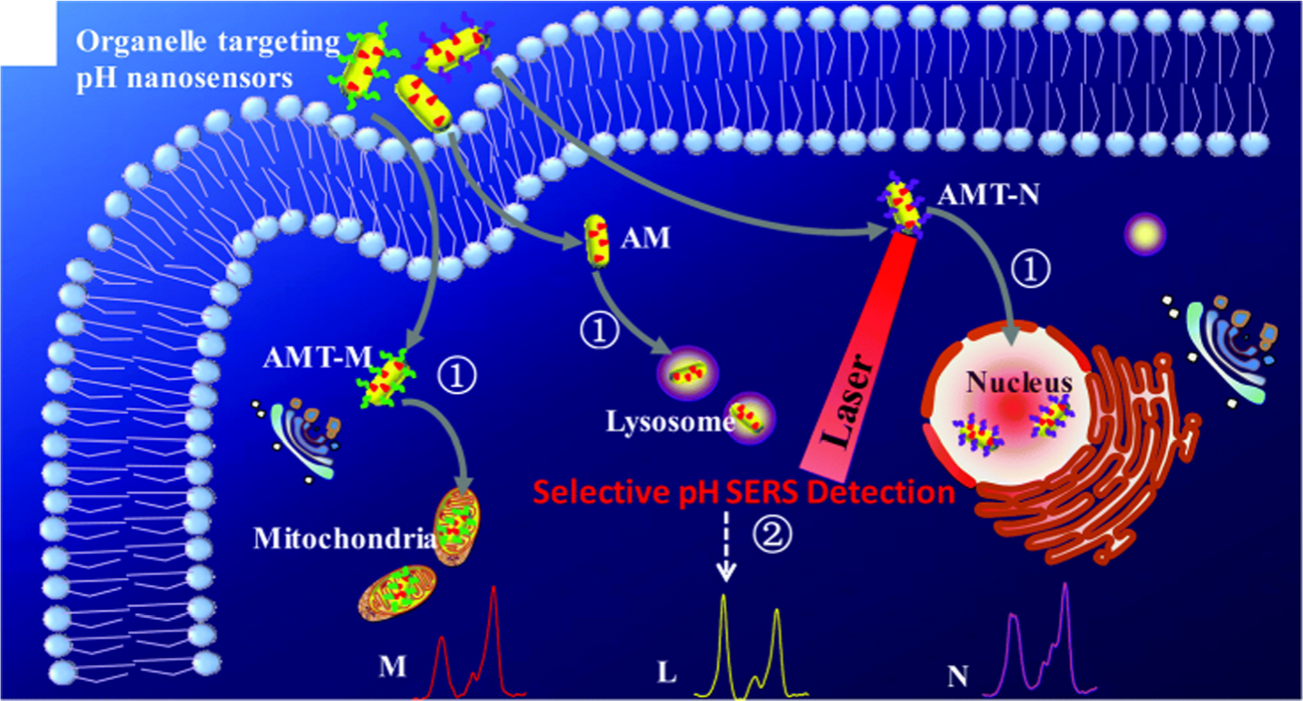
Intracellular pH sensing with targeted AuNP distribution due to a cell-penetrating peptide–mediated method (republished with permission from the Royal Society of Chemistry, from ref [11]; permission conveyed through Copyright Clearance Center, Inc.)

The intra- and extracellular pH (pH_i_ and pH_e_) values of living cells play a major role in the physiology and pathology of diseases. The sensitive and reliable determination of these values could be important for the early diagnosis of many medical conditions. 4-Mercaptopyridine (4MPy) and 4-mercaptobenzoic acid (4MBA) are the two pH sensing molecules mostly used for SERS-based detection [11–15]. They offer the possibility to monitor the pH variations over a large window (pH 4 to 9) and they also show good chemical affinity towards the surface of gold NPs. Generally, NP uptake by living cells is governed by endocytosis and it results in heterogeneous distribution. In order to achieve a targeted uptake, the NPs can be modified with cell-penetrating peptides. Shen et al. prepared gold nanorods functionalized with 4MPy and anchored a nucleus- or a mitochondrion-targeting peptide on their surface (see Fig. 1) [11]. The localization of the SERS tags in the cells was confirmed by super-high-resolution fluorescence imaging and biotransmission electron microscopy. As proof of concept, a tumour and a normal cell line have been investigated. The NPs showed good biocompatibility and the pH value, determined applying the ratiometric approach, of tumour cells showed an acidic character as compared with normal cells.

The reliability of the as-obtained SERS signal is nevertheless often not given. The biggest challenge is brought by the uncontrolled aggregation of the NPs upon uptake. This results in variations of the SERS signal intensity. Bovine serum albumin (BSA) was used to coat Au nanospheres functionalized with 4MPy for reliable pH_i_ sensing in single living cells [12]. To accelerate particle internalization, a cysteine-terminated Tat peptide was also anchored on the NP surface. In a home-built microscopic cell culture platform, the pH_i_ was continuously monitored during the whole cell cycle, including proliferation. This proofs that the NPs have very good biocompatibility and are very promising for live cell investigations. For both studies described above, the NPs that did not enter the cell were removed by washing. Nonetheless, most often not all NPs can be removed in this way. In order to avoid unwanted SERS signals, Bai et al. developed an etchable SERS nanosensor for accurate pH and H_2_O_2_ sensing [13]. Au@Ag core-shell NPs functionalized with BSA and 4MPy were used for this experiment. Upon the addition of an etchant, hexacyanoferrate-thiosulfate, the Ag shell is dissolved and the reporter molecules get detached. Thus, the SERS signal is turned off. Of note, ferricyanide and thiosulfate cannot facilely pass through the living cell membrane and seem not to compromise the cell viability. In this way, pH information will be obtained only from inside of the cell.

Extracellular pH plays an important role in various life processes and it is known to be higher as pH_i_. Two recent studies report on the successful determination of pH_e_ of living cells. One approach uses NPs [14], whereas the second one uses planar SERS substrates [15]. For the first approach, the membrane proteins of cells were biotinylated. This was followed by the addition of streptavidin (SA). The unbound SA was removed from the cell culture and this was followed by the incubation of AuNPs functionalized with 4MBA and biotin. In this way, a sandwich assay was created that assures the presence of AuNPs only on the cell membrane. In the second study, AuNPs deposited on glass were functionalized with 4MPy. The pH_e_ was monitored during cell apoptosis in a home-built cell culture cell.

Cellular gaseous sensing is very challenging, and thus the role of carbon monoxide (CO) or nitric oxide (NO) in cellular processes is not well understood. Similar to the approaches presented above, CO and NO can be also detected via SERS by carefully choosing the reporter molecule that is able to react with the two molecules. In the literature there is only one study reporting on the detection of CO in living cells [16]. The SERS approach was based on the carbonylation of palladacycle (PC) with CO as the carbon source. PC with amino groups were synthesized and assembled on the surface of AuNPs. In the presence of CO, a new band in the SERS spectra was observed making it possible to monitor the carbonylation reaction. Additionally, it was also proven that the sensor shows a high selectivity towards CO. Overall, the AuNP-PC SERS tags are promising for CO cellular monitoring, but curiously no other follow-up publication could be found.

Upon the reaction of *o*-phenylenediamine (OPD) with NO in the presence of O_2_, benzotriazole (BTZ) is generated. Researchers took advantage of this reaction and designed SERS tags to monitor NO in living cells [17, 18]. In a first approach, AuNPs were functionalized with OPD. In the presence of NO, two new bands occur at 789 and 1390 cm^−1^ attributed to NCCN and N=N stretching coupled with ring breathing modes. By applying a ratiometric analysis, good results have been obtained showing excellent selectivity towards NO [17]. In a second publication [18], Au-Ag alloy/porous SiO_2_ core-shell NPs were used as nanoprobes for the ratiometric SERS imaging analysis of NO in living cells. These NPs were chosen because they show good plasmonic properties, are chemically more stable and are less cytotoxic as compared with pure AgNPs, and uncontrolled NP aggregation can be avoided due to the presence of the porous silica shell. These SERS tags were functionalized with 3,4-diaminobenzene-thiol via the SH binding site of the molecule and the OPD reactivity with NO was monitored. As above, a quantitative and specific NO detection in living cells could be achieved.

Besides pH and NO/CO content, the presence of ROS in living cells can give important information regarding the cellular microenvironment. ROS are formed as a natural by-product of the normal metabolism of oxygen and have important roles in cell signalling and homeostasis. As most often these by-products are too small or are not Raman active in order to be directly detected by SERS, molecules that show chemical reactivity with ROS have to be assembled on the surface of the plasmonic NPs. For example hydrogen peroxide was detected using 4-mercaptophenylboronic ester (4-MPBE) [13, 19], hypochlorous acid with 2-mercapto-4-methoxy-phenol (MMP) [20, 21] and peroxynitrate with 4-mercaptophenylboronic acid pinacol ester (MBAPE) [21, 22].

In a recent study, a new approach to detect H_2_O_2_ was reported. Namely, 4-mercaptobenzonitrile (MBN) was immobilized between the Au core and the Ag shell of the SERS tag [23]. MBN shows a sharp and strong band due to the vibration of nitrile in the Raman silent region, and therefore no interference with Raman bands originating from other cellular components takes place. In the presence of H_2_O_2_, the Ag shell is etched and the SERS enhancement is decreased significantly. In order to target the mitochondria, the Au@MBN@Ag NPs were modified with a mitochondrion-targeting peptide. This sandwich nanoprobe was used to monitor ROS during intracellular photothermal therapy. Nonetheless, attention has to be given to the cytotoxic Ag released after ROS reacts with the tags. The cell viability was found to be over 80% at a concentration of 3 nM of nanoprobes.

**Fig. 2:**
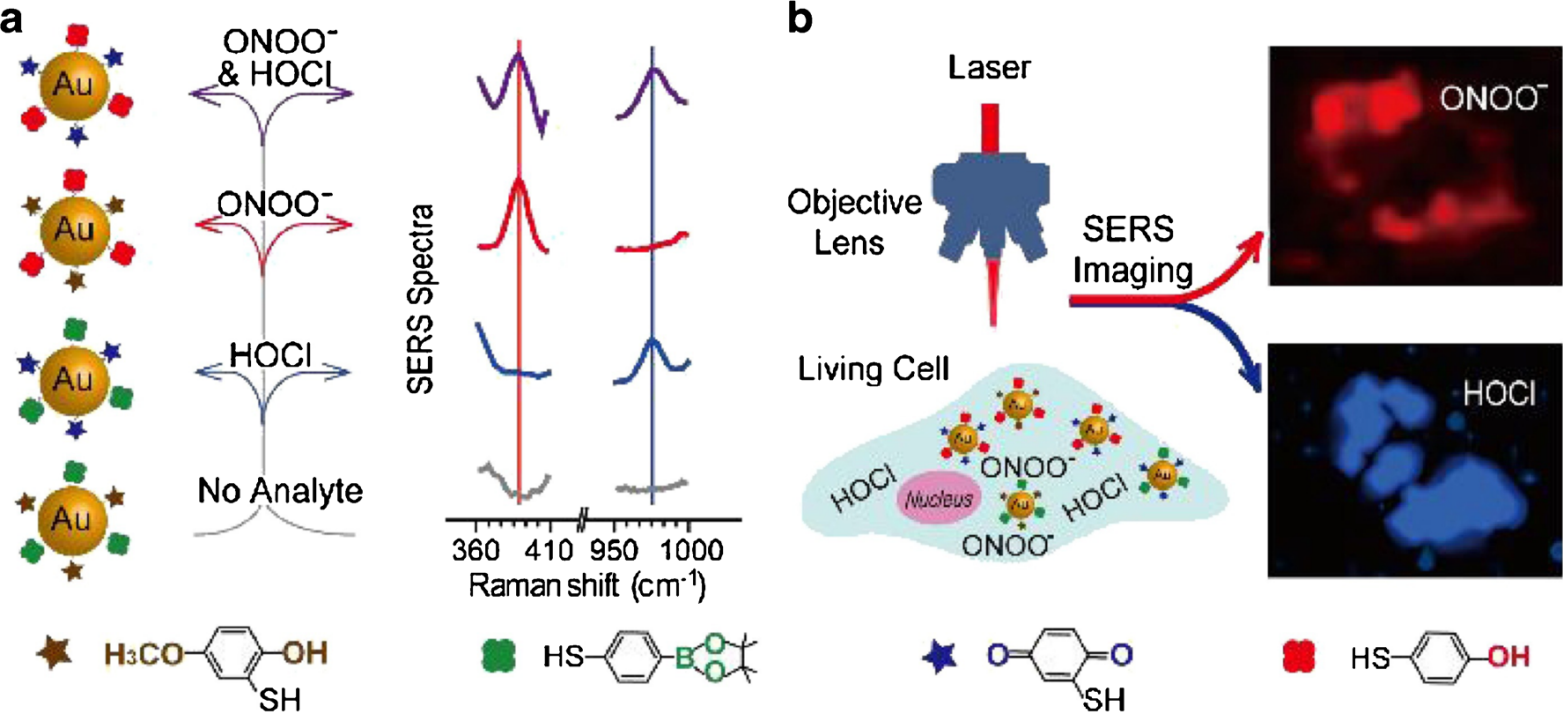
Simultaneous detection of HOCl and ONOO^−^ in living cells using SERS tags. **a** Fabrication and sensing mechanism. **b** Simultaneous SERS imaging of the HOCl and ONOO^−^ in living cells (reprinted from [21], Copyright 2018 with permission from Elsevier)

The simultaneous detection of hypochlorous acid and peroxynitrate in living cells could elucidate diverse physiological and pathological processes. In order to do so, AuNPs were modified with MMP and MBAPE. In the presence of HOCl and ONOO^−^, the hydroxyl and methoxyl moieties of MMP and MBAPE turn into diquinone moieties and phenolic hydroxyl groups respectively (see Fig. 2). This reaction leads to spectral changes in the SERS spectrum of the tags. Due to narrow Raman bands, a multiplex detection was possible with excellent selectivity towards the two targeted ROS. The in situ quantification of HOCl and ONOO^−^ was achieved by a ratiometric approach.

In conclusion, smartly designed SERS tags can access information on the complex processes taking place in living cells that no other technique can, namely, in situ monitoring of pH, gaseous content or ROS during cell proliferation. As described above, different designs of these SERS nanosensors have been investigated. The simplest ones, AuNPs functionalized with sensing molecules although reacting in a specific way with the targeted molecular species, are prone to aggregate and are randomly distributed in the cell. The presence of NP aggregates with different sizes leads to large variations in the SERS signals and yields unreliable signals. To overcome this, the NPs were functionalized with BSA, embedded in a SiO_2_ shell, or planar plasmonic substrates were used. The first two approaches result also in an increased biocompatibility. Furthermore, by modifying the NP surface with cell-penetrating peptides, the uptake of these sensors can be also controlled. Most of the molecular SERS sensors applied in cellular sensing target only one substance. The detection of multiple molecular species is of high interest in order to understand the complex processes. Due to narrow Raman bands and reporter molecules that present Raman bands in the silent region, the task could be fulfilled by talented biochemists. Future research needs to focus on the design of stable, biocompatible nanosensors with multiplexing capability that permit live cell experiments.

## Environmental sensing

The sensing in cellular environment aims to answer fundamental questions, and it is the focus of many academic research institutes. Environmental sensing with molecular SERS sensors, on the other hand, is more application oriented. The final aim is to create accessible commercial analytical platforms that cover existing niches in environmental and food monitoring. Due to their toxicity, the detection of heavy metal ions is of special interest in environmental and food analyses. Techniques used for potentially toxic metal (PTM) quantitation are for example inductively coupled plasma mass spectrometry or atomic emission spectroscopy (ICP-MS/ICP-AES), and they are commonly laboratory based accompanied with high implementation and running costs. Most often, especially, a trained personnel is required to operate such devices. As a consequence, researchers in regions with low infrastructure do not have access to these methods. Therefore, innovative, simple, low-cost and potentially portable techniques are required. Although PTM such as copper, mercury and cobalt are vibrational spectroscopically silent, molecular SERS sensors provide an alternative to the state of the art. Herein, the SERS substrate is functionalized with Raman-active ion-binding ligands. Subsequently, upon heavy metal ion coordination, the vibrational spectrum of the ligand molecule is altered, and ratios in peak intensity and peak shifts are used as indicators of the binding of the target ions.

A target-induced NP self-assembled interface for quantitative Raman detection of Cu^2+^ ions was developed by Yan et al. [24]. In this new approach, core molecule-shell NPs (CMS-NPs) consisting of a gold core with cysteamine and 4MPy bound to the surface are surrounded by a silver shell. The as-prepared self-assembled interfaces were modified with glutathione reporter molecules for detecting copper ions. Thus, a simultaneous detection scheme by bare eyes and SERS was achieved. The bare eye detection was realized down to 10 nM. For SERS detection, the 4MPy molecules between the gold core and the silver shell acted as reporter molecules, reaching a limit of detection (LOD) less than 0.5 nM in water. Cu^2+^ ions could be distinguished from other common metal ions (Mg^2+^, Zn^2+^, Fe^3+^, Pb^2+^, K^+^) with a high specificity.

Recently, Dugandzic et al. realized the detection and quantification of Cu^2+^ ions not only in water but in white wine as well [25]. A dipicolylamine-based ligand was used as a molecular sensor. Dugandzic et al. showed Cu^2+^ ion–specific detection down to 50 nM in water and, furthermore, in a wide concentration range in white wine, achieving an LOD lower than the maximum allowed amount of 7.87 × 10^−6^ M (0.5 μg/mL). Moreover, 4MBA was used as a Raman-sensing molecule for the copper ion detection by Wang et al. [26]. The researchers combined the multiple antibiotic resistance regulator (MarR)-4MBA-AuNP as a SERS detection probe and an anti-Cu^2+^-chelate monoclonal antibody (McAb)-AgNP as a SERS signal amplification probe. Using this dual hot spot model, antigen-antibody interactions cause the combination of AuNP-AgNP heterodimers and the sensitivity of the sensing platform resulted in a LOD of 0.18 nM copper ions in water. The linear range of the response signal was found to be from 0.5 to 1000 nM, which is 5 orders of magnitude lower than the US Environmental Protection Agency limit for Cu^2+^ in drinking water (20 μM). The whole analyses could be completed in less than 15 min. Specificity was shown against 9 other metal ions. The new method was in good agreement with the standard ICP optical emission spectroscopy (ICP-OES) results. The intra-assay coefficient of variation of the SERS sensor was between 3.8 and 7.9%. This indicated acceptable precision and high accuracy of the new SERS assay.

**Fig. 3:**
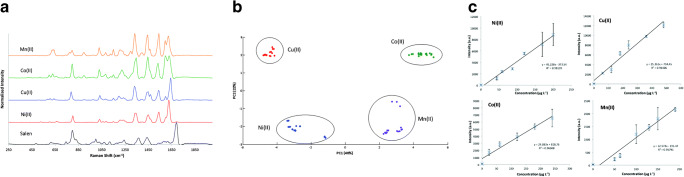
**a** Comparison of the baseline-corrected SERS spectra of the salen complexes studied, using 2.5-μM solutions of each metal ion. Salen, black; Ni(II), red; Cu(II), blue; Co(II), green; Mn(II), orange (*λ*ex = 532 nm; acc. time = 10 s). **b** PCA score plot of the different salen-metal ion complexes. Ni(II), blue; Cu(II), red; Co(II), green; Mn(II), purple. **c** Concentration dependence of each salen-metal complex in real freshwater. Top left: Ni(II) (I_1627_ vs. conc.); top right: Cu(II) (I_1638_ vs. conc.); bottom left: Co(II) (I_1597_ vs. conc.); bottom right: Mn(II) (I_1332_ vs. conc.). Error bars represent the standard deviation between three replicates (*λ*ex = 532 nm, acc. time = 10 s) (republished with permission from the Royal Society of Chemistry, from ref [27]; permission conveyed through Copyright Clearance Center, Inc.)

For multimetal detection, Docherty et al. used a [O,N,N,O] tetradentate bis-Schiff base ligand that was synthesized by reacting salicylaldehyde with 1,2-diaminoethane [27]. With this ligand, named salen, the researchers were able to detect Ni^2+^, Co^2+^, Cu^2+^ and Mn^2+^. Each metal-salen complex results in significantly different SERS spectra. Thus, the unique spectral shape could be used to identify which metal ion was present. Remarkable changes between each of the complexes were recognized for the two peaks around 1600 cm^−1^. These two bands are assigned to the C=N stretching of Schiff bases. For Cu^2+^, a strong band is observed at 1641 cm^−1^ with a weaker band at 1597 cm^−1^. However, for Co^2+^ ions, this band shifts to 1628 cm^−1^ and 1597 cm^−1^. For Ni^2+^ and Mn^2+^, these bands again significantly shifted to 1628 cm^−1^, with a shoulder at 1600 cm^−1^ for nickel ions, and to 1621 cm^−1^ and 1597 cm^−1^ for mangan ions, respectively (see Fig. 3a). These bands were assigned to the C=N stretch of salen. Due to the changes induced by the binding of different metal ions to the nitrogen atoms of the Schiff base, the detected position of the bands around 1600 cm^−1^ was altered. Thus, these peaks were used as marker bands for the identification and quantitation.

Applying the principal component analysis (PCA), the researchers proved that the differences were significant (see Fig. 3b). Furthermore, Ni^2+^, Cu^2+^ and Mn^2+^ can be detected below the WHO’s recommended limits (0.02 mg/L, 2 mg/L, 0.5 mg/L, respectively) at concentrations of 0.001, 0.002 and 0.002 mg/L, respectively. It could be shown that the method is reasonably comparable with the ICP-MS analysis. Furthermore, clear tap water was successfully tested for the four metals (depicted in Fig. 3c). This indicates that the presented method might be capable of detecting metal ions in contaminated water samples.

**Fig. 4:**
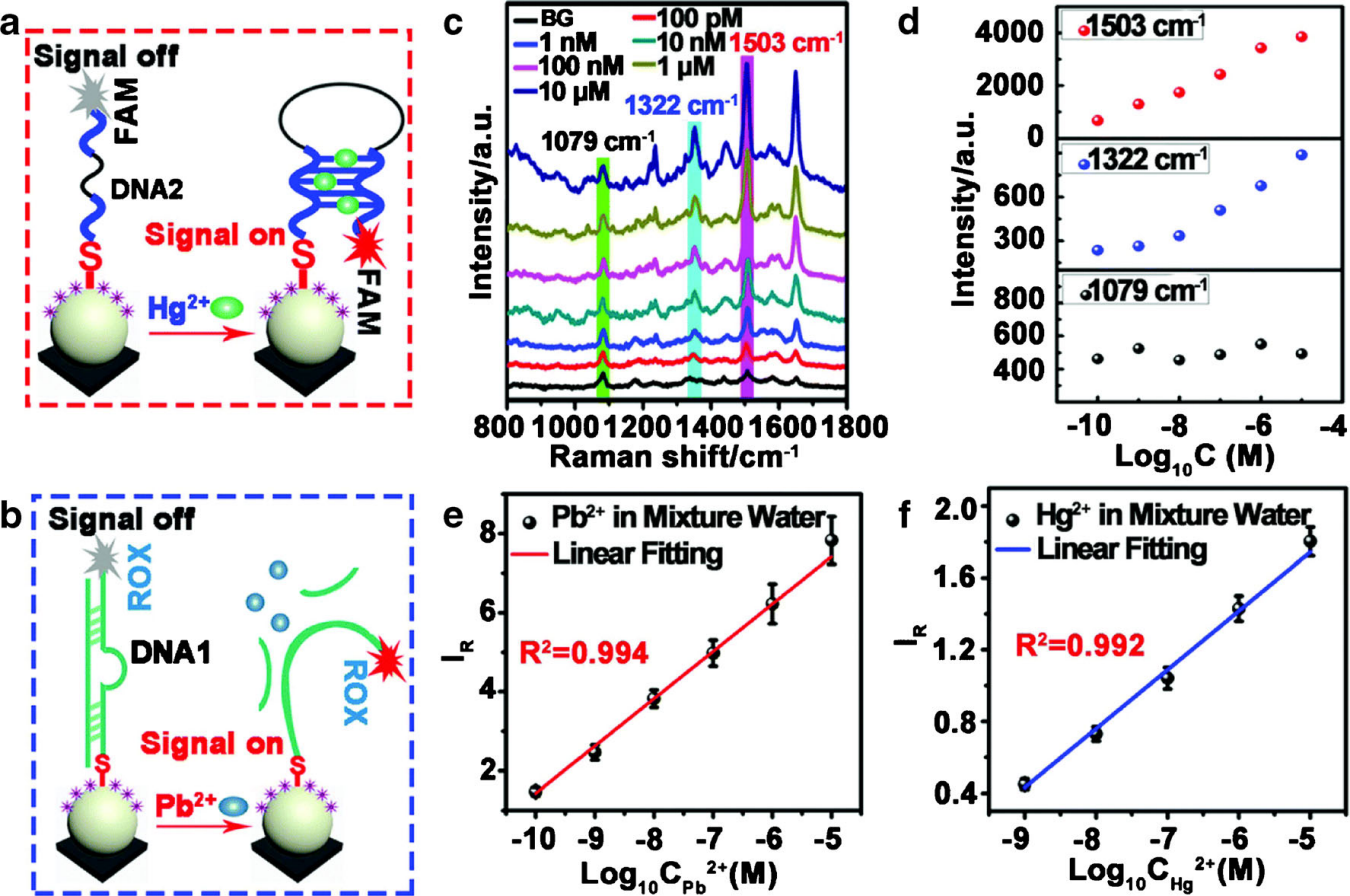
**a** Schematic of IS-Si@Ag NPs for Hg^2+^ and **b** for Pb^2+^detection. **c** SERS spectra of the functionalized silicon SERS chip in the presence of Pb^2+^ and Hg^2+^ with the same concentrations ranging from 100 pM to 10 μM. Background (BG) stands for the distilled water. **d** Corresponding plots of the SERS intensities of the 4-ATP (1079 cm^−1^), FAM (1322 cm^−1^) and ROX (1503 cm^−1^). **e** The linear fitting of the SERS relative intensities (IR = I_1503_/I_1079_) versus the logarithmic Pb^2+^ concentration (1.0 × 10^−10^ to 1.0 × 10^−5^ M). **f** The linear fitting of the SERS relative intensities (IR = I_1322_/I_1079_) versus the logarithmic Hg^2+^ concentration (1.0 × 10^−9^ to 1.0 × 10^−5^ M). All error bars show the standard deviation determined from three independent assays. Excitation wavelength, 633 nm; laser power, 0.2 mW (republished with permission from the Royal Society of Chemistry, from ref [28]; permission conveyed through Copyright Clearance Center, Inc.)

For the simultaneous detection of lead (Pb^2+^) and mercury (Hg^2+^), Shi et al. developed silicon SERS chips including an internal standard sensing strategy [28]. For the detection in industrial wastewater, a silicon wafer was coated with AgNPs as an internal standard and the NPs were modified with 4-aminothiophenol (4-ATP). Pb^2+^/Hg^2+^-responsive DNA strands were conjugated to the chip. For sensing Hg^2+^, the marker was HS-B1-FAM (single-stranded DNA—ssDNA—labelled with FAM (carboxyfluorescein), containing consecutive thymine (T) bases), while for Pb^2+^, a double-stranded DNA (dsDNA) labelled with ROX (6-carboxy-X-rhodamine), specific for Pb^2+^ (HS-17E-ROX), was used (depicted in Fig. 4a and b, respectively). The SERS spectra specific for Pb^2+^ and Hg^2+^ ions are shown in Fig. 4c with the same concentrations ranging from 100 pM to 10 μM. The characteristic SERS band was found at 1322 cm^−1^ for FAM and at 1503 cm^−1^ for ROX, respectively, as shown in Fig. 4d. The marker band of 4-ATP (1079 cm^−1^) was used for normalization. Thus, the LOD was found to be 99 pM (19.8 ppt) for Pb^2+^ and 0.84 nM (168 ppt) for Hg^2+^, respectively (see Fig. 4e, f). For Hg^2+^, this is around two orders of magnitude lower than the value defined by US EPA (10 nM, 2 ppb). The specificity of this SERS application was investigated by mixing twelve metal ions (i.e., Zn^2+^, Ni^2+^, Na^+^, Mn^2+^, Mg^2+^, Fe^2+^, Cu^2+^, Co^2+^, Ca^2+^, Ba^2+^, Pb^2+^, Hg^2+^) and the mixed samples containing Pb^2+^ and Hg^2+^ at a concentration of 100 nM. Out of these twelve tested metals, only Pb^2+^ and Hg^2+^ exhibit strong SERS responses at 1503 cm^−1^ or 1322 cm^−1^, respectively. Distinct SERS signals were observed at both 1503 cm^−1^ and 1322 cm^−1^ for the mixture containing both Pb^2+^ and Hg^2+^ ions. To demonstrate the application, the developed chip was combined with a portable Raman microscope and employed for the simultaneous quantitative detection of Hg^2+^ and Pb^2+^ in industrial wastewater. Thereby, the achieved relative standard deviation values were less than 15%.

Another highly sensitive dual-responsive optical marker for mercury ions in drinking water was developed by Makam et al. [29]. The sensor based on histidine (H)-conjugated perylene diimide (PDI) bolaamphiphile (HPH) supported on gold-deposited monodispersed nanosphere monolayers (Au-MNM) of polystyrene. A remarkably LOD value of 60 aM (0.01 parts per quadrillion (ppq)) for Hg^2+^ in water was achieved analysing the SERS signal of HPH. This is 9 orders of magnitude lower than the US EPA tolerance limit of Hg^2+^ in drinking water (10 nM, 2 ppb). At the same time, the system provides highly selective and sensitive visible Hg^2+^ fluorescence–based detection (sol-to-gel transformation), with detection limit of 5 nM (0.1 ppb). The assay was also tested for the selectivity of the probe HPH. Thus, several alkali metal and transition metal perchlorate salts were tested. The sensor did not show any significant fluorescence changes in the presence of most of the metal ions (Mn^+^: Cu^2+^, Cd^2+^, Co^3+^, Fe^3+^, Mg^2+^, Pb^2+^, Ca^2+^, Zn^2+^, K^+^, Na^+^, Ni^2+^, Cr^3+^, Ag^+^, Al^3+^ and Fe^2+^) tested in water. The preferential selectivity of HPH towards Hg^2+^ could be proven.

For a selective and quantitative detection of trace amounts of mercury and copper, Tang et al. functionalized gold nanorod (AuNR)-polycaprolactone nanocomposite fibres with 2,5-dimercapto-1,3,4-thiadiazole (di-DMT) for Hg^2+^ and trimercaptotriazine (TMT) for Cu^2+^ sensing, respectively [30]. Concentrations down to 1 μM were tested and a linear range was defined for signal intensity vs. concentration from 10 mM to 1 μM for Hg^2+^. While for Cu^2+^, the linear range was defined from 100 to 1 μM only. Although the sensitivity was comparably weak, in this study, the authors could prove very high selectivity using Cu^2+^, Zn^2+^, Cd^2+^, Pb^2+^ and Hg^2+^ for both applied bridging molecules, di-DTM and TMT, respectively.

In water and environmental analyses, not only metal ions are of special interest but pesticides and environmental hazards are under investigation as well. One example is the indirect SERS detection of tetrabromobisphenol A (TBBPA) [31]. The hydrophobic analyte TBBPA was detected in water with a reproducible LOD of 10 pM. For the presented indirect detection, magnetic gold nanoclusters (MGNCs) were functionalized with 4-dimethylaminopyridine (DMAP). The authors stated that the modulation of the DMAP SERS spectrum was specific to the analyte. Thus, the method could be applied to other hydrophobic analytes, each with its own unique spectral identity.

The well-known human carcinogen trichloroethylene (TCE) was detected by Yu et al. using self-referenced SERS and gold core/silver shell NPs containing 4-mercaptophenylboronic acid (4-MPBA) between the core and shell as an internal reference (Raman peak at 534 cm^−1^) [32]. Due to the so-called Fujiwara reaction (TCE reacts with 4-MPy), the presence of TCE in water causes the consumption of 4-MPy. This results in a change in the intensity of the Raman signal of 4-MPy at 1220 cm^−1^. A linear concentration dependency was found for the range from 0.2 to 1.0 μM, and the LOD was determined to be as low as 8 ppb (60 nM). The researchers could prove that this method is sensitive to TCE (compared with Cu^2+^ ions, phenol and toluene after the Fujiwara reaction). Furthermore, TCE was successfully detected in spiked lake water (concentrations 0.3 and 1 μM), showing the robustness of the approach.

In summary, the application of molecular SERS sensor in environmental monitoring is often associated with the detection of metal ions. The first studies reported on this topic concentrated on single-metal detection in pure water samples. By smart design of the SERS particles, probes suitable for the simultaneous and specific detection of multiple species were achieved. The investigated sample matrix gained complexity over time; i.e., metal ions in industrial wastewater were detected. The results presented above are all in the proof of concept stage. While cellular environment sensing represents fundamental research, the detection of metal ions in the environment has an applicative character. In the future, efforts need to be directed towards proving the robustness of this technique and to transfer the knowledge from the lab to the field.

## Outlook

**Scheme 1:**
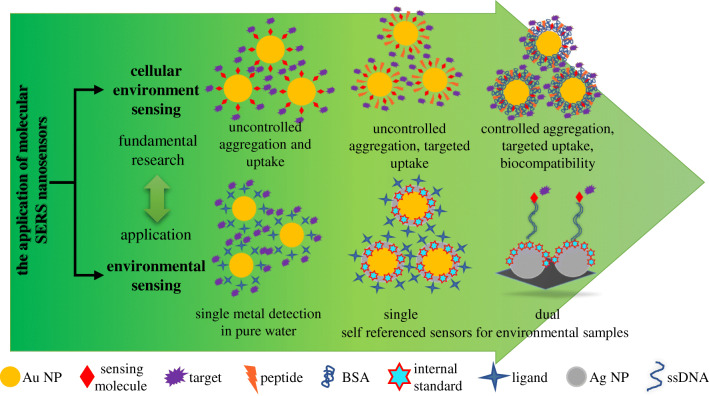
The evolution of the applications of molecular SERS nanosensors

Within this trend article, we present recent results on the SERS-based detection using molecular sensors as monitoring platforms. The increasing number of papers published on this topic is a clear proof of the importance of this field. However, SERS as an analytical method, even after almost half of century since its discovery, is still not commercialized or widely applied in clinical laboratory. The parameter which plays a major role in this is the SERS active substrate. As described above, SERS sensors evolved considerably, making the sensitive and specific in situ detection of very small molecules possible (see Scheme 1). However, the reproducibility of synthesis and long-term stability of these sensors are still challenging. There are a few commercial SERS substrates available, and they are mostly produced only out of gold and rarely are functionalized with reporters or recognition elements. Another challenge one faces when performing SERS measurements is the way the sample is applied/mixed with the SERS active substrates. In order to have a robust technique, a considerable attention has to be given to this aspect. Furthermore, reliable controls that test whether the sensor is still working have to be thought about. This aspect is very seldom addressed in SERS studies. Lastly, data analysis does not need to be underestimated. Erroneously applied multivariate statistical methods can lead to results that are just apparently right. In our opinion, once these aspects are addressed, SERS could offer great solutions for application fields where at the moment cost-intensive methods are used. The development of portable and miniaturized Raman systems has led already to many user-friendly and cost-effective devices.
